# Out of Control – Privacy Calculus and the Effect of Perceived Control and Moral Considerations on the Usage of IoT Healthcare Devices

**DOI:** 10.3389/fpsyg.2020.582054

**Published:** 2020-11-11

**Authors:** Evgenia Princi, Nicole C. Krämer

**Affiliations:** Social Psychology: Media and Communication, Department of Computer Science and Applied Cognitive Science, University of Duisburg-Essen, Duisburg, Germany

**Keywords:** IoT, control, privacy calculus, pervasive healthcare, eHealth, moral considerations

## Abstract

People are increasingly applying Internet of Things (IoT) devices that help them improve their fitness and provide information about their state of health. Although the acceptance of healthcare devices is increasing throughout the general population, IoT gadgets are reliant on sensitive user data in order to provide full functioning and customized operation. More than in other areas of IoT, healthcare applications pose a challenge to individual privacy. In this study, we examine whether actual and perceived control of collected data affects the willingness to use an IoT healthcare device. We further measure actual behavior as a result of a risk-benefit trade-off within the framework of privacy calculus theory. Our experiment with *N* = 209 participants demonstrates that while actual control does not affect the willingness to use IoT in healthcare, people have a higher intention to use an IoT healthcare device when they perceive to be in control of their data. Furthermore, we found that, prior to their decision, individuals weigh perceived risks and anticipated benefits of information disclosure, which demonstrates the potential to apply the privacy calculus in the context of IoT healthcare technology. Finally, users’ moral considerations of IoT in healthcare are discussed.

## Introduction

Smart technologies that are connected in the Internet of Things (IoT) have long found their way into our everyday lives. IoT devices such as smart TVs or robotic vacuum cleaners are an integral part of many households or, as it is the case with smart fitness trackers, our constant companions while they collect, process and save user data. For this purpose, IoT applications are equipped with various sensors that for example enable geolocation, measure movement or record sound or video (Swan, 2012). What makes these devices ‘smart’ is their ability to interact over networks, sense, modify behavior and draw conclusions from rules. The underlying functionality of such devices is based on an extensive collection of user data, which is merged with other information available about the individual. The automated combination and analysis of the data in turn not only serves as a template, e.g., for personalized services but is also a possibility to predict our behavior (Flores-Martin, 2017). Accordingly, extensive user profiles are of enormous importance and many companies and institutions have a great interest in gaining access to these profiles (Marreiros et al., 2017; Romanou, 2018), e.g., to better respond to individual customer needs and thus to prevail in the highly competitive IoT market or, as in the case of COVID-19, to have access to extensive data that lead to more accurate and anticipated research results. In order to provide directives and define minimal standards for the extensive collection of private data as well as to protect the privacy of individuals, the EU has passed the General Data Protection Regulation (GDPR). This legislation, among others, requires informed consent of the user toward data collection and guarantees access to information about what data was gathered and what was the purpose of data processing. However, the realization of these requirements is often problematic due to the lack of transparency (Kröger, 2018; Rantos et al., 2018) and because people want to benefit from the promising technology.

Especially in the healthcare sector, substantial technical development has been achieved due to IoT. The integration of heterogeneous sensors in medical IoT applications enables, among other things, the monitoring of body temperature, non-invasive glucose sensing for patients with diabetes or the recording of oxygen saturation (Islam et al., 2015). Various vital signs are tracked, automatically analyzed and forwarded to the treating physicians in real time. This offers enormous advantages in the treatment of pediatric and elderly patients or people with chronic diseases as this monitoring enables individuals to be more independent, for example by reducing the need for hospitalization or delay the move to the retirement home (Mittelstadt, 2017). However, while benefits of IoT within the electronic Health (eHealth) are particularly high, user privacy might be increasingly violated due to data sensitivity. Many IoT applications that are used in eHealth collect and store personal health records, which among other things include existing disorders or physical/health-related parameters (Doukas and Maglogiannis, 2012). According to the Privacy Calculus Theory (Culnan and Armstrong, 1999), users of such applications are faced with a decision either to provide access to their data in order to benefit from the technology or not to reveal any information to avoid the risk of data mishandling and violation of their privacy. As a result, the trade of personal data is fueled, which means that the secure handling of user data can no longer be guaranteed. While access to data obtained by biometrical sensors or microphones requires explicit user consent, data collected from sensors which are considered harmless (e.g., accelerometers, temperature or light sensors) can be often accessed by third parties without security permission (Kröger, 2018) still allowing inferences for example about the users’ environment. Thus, there is a lack of transparency regarding data collection by IoT, which makes it difficult for users to understand what happens to their data – whether it is stored permanently, processed or even passed on to third parties. This underscores the need to gain control over the handling and accessibility of these data (Porambage et al., 2016). In our work, based on the finding that an overwhelming majority of American consumers stated in a survey that they had lost control over their data (Gerber et al., 2018), we aim to shed more light on the role of actual and perceived control on personal data collected by IoT in healthcare. As the adoption of IoT in healthcare entails both great potential and a possible threat for privacy, another contribution of this paper is to investigate in more detail whether the privacy calculus theory (Culnan and Armstrong, 1999) is a suitable theoretical approach in the context of smart technology deployment in healthcare. To the best of our knowledge, no one so far has examined this risk-benefit trade-off as theoretical basis for IoT usage in eHealth measuring actual behavior.

Finally, in this study moral considerations of users are discussed, as the adoption and growing use of smart technology in healthcare is changing fundamental social norms and values. Automated processing and analysis of patient data by means of technology or access to health information by health insurance companies could be used for classification of patients into risk groups or different tariffs. This, in turn, could raise moral concerns among those affected. Employers might also demand access to health data of applicants or employees with potential disadvantage of sick people, which means that the broad adoption of IoT could have negative effects on solidarity within society. Therefore, we further examine moral attitudes as an influencing factor of the intention to use IoT in eHealth.

## Literature Review

### IoT in Healthcare

One of the areas in which IoT is making great strides, both in development and deployment, is eHealth. The establishment of various IoT systems in this sector will bring about profound changes for all parties involved, such as patients, attending physicians, healthcare institutions and health insurance companies, thus revolutionizing the healthcare system. Smart technologies are designed to help reduce costs, improve the quality of life of patients and simplify medical care by practitioners (Catarinucci et al., 2015). There is a wide range of possibilities of IoT-usage in this area, just as the recent approach to evaluate data tracked by smartwatches in order to tackle COVID-19 as well as other health-related aspects. Nausheen and Begum (2018) distinguish three areas of application for IoT when it comes to the implementation of information and communication technology in medical care: (I) mobile health applications (mHealth), (II) wearable health gadgets and (III) internal medical devices. mHealth (I) comprises electronic solutions on mobile devices. These include, for example, apps operating on smartphones. These can be calculator apps that compute certain parameters such as calories consumed and burned using formulas or diagnostic apps, which provide individuals with treatment information. Wearable healthcare devices (II), such as a wrist band, are equipped with multiple sensors and either directly display information about steps taken and heart rate or transmit this information to a smartphone. Furthermore, by means of such devices it can be determined in real time whether a person has fallen (e.g., by combining GPS and accelerometer data) so that help can be requested immediately and autonomously with an integrated alert function. In addition, commercial healthcare products are also used in order to monitor one’s own fitness, for example by quantifying one’s nutrition or sleep. Internal medical devices (III), such as a smart pacemaker, describe a further technological advancement. These devices can be implanted into the human body either transmitting data or automatically performing actions, for example, by releasing insulin when the blood glucose level is too high. Thus, operations could be avoided, and life-threatening risks might be reduced, which represents the enormous potential of using IoT devices and applications in eHealth. Since the focus of this study is on IoT technology, the area of mHealth and health apps is excluded. Instead, we investigate the use of a portable IoT device for explicit deployment in healthcare. Because internal medical devices are used by only a comparatively small number of people, we choose a device that is relevant to the everyday life of the general population. In the methods section the device is described in more detail.

However, in no other area in which IoT is utilized the impact on user privacy is as high as in medical and health care (Laplante and Laplante, 2016). In order to enable remote diagnosis and to propose individual therapies based on the analysis of extensive data, comprehensive patient records must be generated, which can be accessed by all involved parties in real time. The person-related information contained in these patient records or captured by mobile, wearable or internal devices is highly sensitive, giving cause to a real privacy threat (Mittelstadt, 2017).

### Privacy Calculus

When talking about the use of IoT in healthcare, the foregoing explanations indicate that the pivotal aspect of user privacy must be taken into account. Due to the sensitivity of the data that needs to be tracked for the full functionality of specific IoT applications, as well as other data collected for machine learning purposes, for example, it is inherent that user privacy is severely compromised. However, as the preceding statements show, IoT brings tremendous opportunities, especially in the area of healthcare (e.g., telemedicine or prevention of surgical procedures). Consequently, the user must weigh up the benefits he or she expects from the use of an eHealth device and the risks associated with a potential privacy violation.

This balancing process is described by the privacy calculus theory (Culnan and Armstrong, 1999; Dinev and Hart, 2006). The theory is based on the assumption that individuals evaluate anticipated benefits and perceived risks in order to make a rational decision regarding the disclosure of their personal data.

The application of privacy calculus originates from eCommerce (Dinev and Hart, 2006) and was later extended to the use of websites and social networking sites (Krasnova et al., 2012; Bol et al., 2018). The deployment of IoT extends the potential threat for privacy by tracking data not only in the virtual world but also within the physical environment of the user. Therefore, more recently, privacy calculus has been applied to the field of IoT, where researchers have shown that in addition to online data sharing, advantages and disadvantages are weighed before users provide personal information when deploying IoT (Lee et al., 2018; Wiegard et al., 2019).

Privacy calculus has been criticized by some scholars, stating that decisions of individuals might be restricted by behavioral biases (Acquisti and Grossklags, 2005) such as overconfidence (Brandimarte et al., 2013). Furthermore, privacy decisions are usually based on incomplete information (Marreiros et al., 2017) and even if detailed information is available the ability of individual’s cognitive processing might be limited resulting in an imperfect decision. However, Gerber et al. (2018) argue that this decision “might therefore seem irrational to an external observer, but at the same time fairly rational to the decision maker” (p. 6). Results of numerous studies in different contexts additionally provide strong evidence for the privacy calculus model explaining privacy-related behavioral intention and actual behavior (Krasnova et al., 2012; Dienlin and Metzger, 2016; Kim et al., 2019).

As determinants for the privacy calculus, perceived risks and anticipated benefits of information disclosure were examined in numerous studies. The results of Kim et al. (2019) demonstrated that both perceived benefits and perceived privacy risks have an effect on the willingness to provide personal information when using different IoT services. In their study, Princi and Krämer (2020) showed that anticipated benefits of household IoT in private environments are a decisive factor for the intention of their usage. Due to the great potential of IoT specifically in healthcare, scientific interest is also increasing with regard to eHealth. Karahoca et al. (2018) demonstrated the significant positive effect of perceived benefits on the intention to adopt IoT healthcare technology products. Other studies postulate the trade-off between privacy and functionality, as more data enables better functioning and a broader range of possibilities for the implementation of IoT in healthcare (Laplante and Laplante, 2016). Mittelstadt (2017) further states that “These basic functions may improve healthcare through increasingly granular monitoring and personalized interventions (…), yet they simultaneously create an opportunity for violating user expectations of personal and informational privacy.” (p. 160) illustrating the balancing act.

Although these studies substantiate the appropriateness of privacy calculus theory in the area of IoT in healthcare, there is a lack of research on actual user behavior, as so far only the intention to use IoT and willingness to disclose data have been addressed. Due to the self-reporting method, behavioral intention is only of limited suitability to predict actual behavior (Young et al., 1998). Therefore, we investigate the privacy calculus measuring actual behavior of individuals with regard to the usage of an IoT healthcare device. Based on results of the aforementioned literature (Karahoca et al., 2018; Kim et al., 2019; Princi and Krämer, 2020), we assume that specific benefits people anticipate from IoT in eHealth lead to a higher usage of respective IoT devices. As in line with privacy calculus assumptions, these benefits are opposed to privacy related risks, meaning “the expectation of losses associated with the release of personal information” (Xu et al., 2011, p. 46), we further conclude that people will rather not use IoT in healthcare when they perceive privacy risks:

Hypothesis 1 (*H1*): Perceived benefits of an eHealth device have a positive effect on the confirmation of actual use of the device.

Hypothesis 2 (*H2*): Perceived privacy risks of an eHealth device have a negative effect the confirmation of actual use of the device.

### Perceived Control of Private Data

A fundamental aspect of privacy is the control over personal data. Westin (1967) states that privacy is “the claim of individuals, groups, or institutions to determine for themselves when, how, and to what extent information about them is communicated to others” (p. 7). Similarly, Altman’s (1975) definition of privacy is “selective control of access to the self or to one’s group” (p. 18). This implies that control over personal data and its utilization and handling must be provided to users in order to ensure privacy.

Perceived control is also one of the cornerstones of the theory of planned behavior (Ajzen, 1991). This theory states that behavioral intentions and subsequently actual behavior of individuals is a reflection of the interplay of their attitudes, perceived social norms and perceived control over an action. At this point it is necessary to distinguish between actual and perceived control. Particularly in eHealth, actual control can be established, for example, by means of technical default settings. Sensitive data, for instance, can be locally anonymized prior to transmission (Clarke and Steele, 2015). However, many studies show that the amount of control actually given in a situation can differ from individually perceived control (Ajzen, 1988; Bugental et al., 1989). As argued by Skinner (1996), “The most fundamental distinction in the literature on control is between actual control, or the objective control conditions present in the context and the person, and perceived control, or an individual’s beliefs about how much control is available.” (p. 551). This means that users who, for example, are unable to apply provided control tools will not have the feeling of being able to control their data, despite the objectively available control. At the same time, in an objectively uncontrollable situation, people might still believe that they are in control, which is sufficient to trigger actions and stimulate arousal (Averill, 1973). Furthermore, control creates a sense of security and is a crucial factor in assessing potential risks (Slovic, 1987). Therefore, in the era of digital information, where data are the means of payment and companies and other institutions have a great interest in obtaining these information (Romanou, 2018), control over private data is more important than ever. Wang et al. (2015) state that control over the purpose of information collection determines users’ provision of personal data. Brandimarte et al. (2013) further found that individuals disclose even identifiable information when they perceive to be in control over its release and access. Users of IoT, who believed they could control which data were collected, were also willing to share their data with third parties (Beales and Muris, 2019).

Based on these elaborations, we assume that providing individuals with possibilities to control their data will positively affect their intention to use IoT devices in healthcare. We further test for a positive effect of perceived control on the intention to use. Given the correlation between the intention to engage in a behavior and its actual performance postulated by the theory of planned behavior (Ajzen, 1991), we expect that the intention to use will positively affect actual usage of an eHealth device. Therefore, we formulated the following hypotheses:

Hypothesis 3 (*H3*): Participants’ intention to use an eHealth device will be higher if they are provided with actual control of private data.

Hypothesis 4 (*H4*): Perceived control of private data has a positive effect on the intention to use an eHealth device.

Hypothesis 5 (*H5*): Participants’ intention to use an eHealth device will have a positive effect on the confirmation of actual use of the device.

Furthermore, perceived control leads to a lower assessment of risks (Slovic, 1987) and a more positive attitude toward the disclosure of personal data, as users are less concerned about the collection of their data (Hajli and Lin, 2016). Therefore, we also assume that users of eHealth devices will perceive fewer risks and more benefits when they perceive control:

Hypothesis 6 (*H6*): Perceived control of private data has a positive effect on the perception of benefits of an eHealth device.

Hypothesis 7 (*H7*): Perceived control of private data has a negative effect on the perception of privacy risks of an eHealth device.

### Privacy Concerns

The ability of IoT technology to collect and aggregate extensive user data and, at the same time, media-effective scandals of privacy breaches and constant data leaks create uncertainty among users and concerns regarding the privacy of their data: how secure are my data? What data are collected, what is the purpose and who is authorized to access it? Where are the data stored and how long do they remain on servers? These privacy-related questions often remain unanswered. This is partly due to the lack of transparency of manufacturers and providers of applications (Rantos et al., 2018). On the other hand, it is simply not feasible to retain an overview of the diverse applications of IoT in everyday life, accompanied by varying regulations regarding usage and protection of data from different companies (Acquisti et al., 2015; Marreiros et al., 2017). Furthermore, devices from the same category are equipped with different sensors and may therefore differ in their mode of operation and data tracking (Swan, 2012; Porambage et al., 2016). In this respect, privacy concerns should not only be based on the amount of information that can be collected about the user by different IoT applications, but above all on the conclusions drawn about the behavior, preferences and habits of users that can be inferred by means of algorithms analyzing these data (Zheng et al., 2018).

Against the background of the monetary value that can be attributed to inferred information of health-related data (Mittelstadt, 2017), stakeholders, such as health insurance companies or employers, might also be interested in this data, increasing further privacy concerns. When individuals fear that their health conditions will prevent them from getting a job or lead to higher health insurance rates (i.e., price discrimination), it can be assumed that they are likely to have higher concerns regarding disclosure of their health-related data.

However, privacy concerns do not necessarily have a direct influence on the disclosure of personal data. Many studies have revealed that individuals showed disclosing behavior and used applications which require sensitive data despite expressions of privacy concerns (Acquisti and Grossklags, 2005; Barnes, 2006; Norberg et al., 2007; Egelman et al., 2013; Taddicken, 2014). This discrepancy was referred to as the Privacy Paradox (Barnes, 2006; Norberg et al., 2007). And although this approach has been resolved (Dienlin and Trepte, 2015; Gerber et al., 2018), e.g., by the theory of planned behavior (Ajzen, 1991), there is no consensus within the scientific community about the exact role of privacy concerns. Some studies indicate direct effects of privacy concerns on user behavior, such as the negative influence on the willingness to provide personal information (Dinev and Hart, 2006). Privacy concerns are therefore negatively associated with disclosing behavior (Gerber et al., 2018). In contrast, Yun et al. (2013) provide evidence that privacy concerns moderate the adoption of location-based services which track users’ GPS data. Ioannou et al. (2020) in turn refer to the contextual dependence of privacy concerns with regard to the disclosure of biometric data. Against this background, we ask which role individuals’ privacy concerns take in the deployment of IoT devices in healthcare by formulating the following research question:

Research question 1 (*RQ1*): Do privacy concerns have a negative effect on the intention to use an eHealth device?

### Moral Considerations of eHealth Users

Along with opportunities, privacy concerns and potential risks of IoT applications, ethical issues have been addressed by many scholars as a major challenge of IoT deployment (Habib, 2014; Hajli and Lin, 2016; Mittelstadt, 2017; Allhoff and Henschke, 2018). The extensive application of networked technologies in almost all areas of everyday life inevitably leads to changes in social norms and values (Allhoff and Henschke, 2018). In addition to fundamental discussions regarding actual utilization of technology in healthcare (Fleming et al., 2009) also legal questions arise, for example, regarding who is responsible for the failure of machines or the loss of sensitive data (AboBakr and Azer, 2018).

Thus, studies in this field examine ethical cornerstones of eHealth and their societal role rather than the personal attitude of individuals regarding morality in this sector. Though we acknowledge the profound relevance of ethical challenges, in our work we focus on the role of moral considerations of IoT users. Specifically, we are interested in whether the moral attitude of an individual has an effect on his or her intention to use IoT in healthcare. In this regard, van den Hoven (2017) emphasizes the importance of fundamental human moral values, such as equity and justice by stating that “Human beings, whether in their role as employers, consumers, citizens, or patients, have moral values, moral preferences and moral ideals. Information technology cannot and ought not to be at odds with them, and preferably should support and express them” (p. 67). He further associates the social acceptance of these technologies with the extent to which the technologies incorporate moral values of customers. In this context, ethical decision-making models assume that individuals include moral aspects in their decisions and act according to their moral considerations (Rest, 1986; Stadler, 1986). Therefore, the suggestion seems plausible that people are more likely to use IoT in eHealth if it corresponds to their moral concepts.

In the health sector, moral concerns could relate, for example, to the digital divide, i.e., whether the introduction of the technology would result in only privileged groups having access to it and benefit from its advantages, or whether the consequences of its deployment would be morally acceptable. Such consequences could include price discrimination against customers of health insurance companies. Particularly these institutions have a legitimate interest in the health data of patients tracked and analyzed by IoT, because so-called risk patients cause billions of euros in costs every year (Neaţu, 2015). Thus, it is not unreasonable to consider discriminatory profiling or manipulative marketing as a result of accessibility to more information (Montgomery et al., 2018). Health insurance companies, which receive patient data indicating, among other things, obesity, regular high blood pressure and acute lack of physical activity, might classify these patients in more expensive tariffs or possibly not insure them at all. This kind of approach would mean enormous savings potential for the health insurance companies. At the same time, it would be morally questionable, as certain groups of people (e.g., people with previous illnesses) would be discriminated against, contradicting the idea of a solidarity-based society. Given this discrepancy, our work reflects on the moral attitude of individuals and its effect on the willingness to use IoT in healthcare. Therefore, we test the following assumption:

Hypothesis H8 (*H8*): Moral considerations have a negative effect on the intention to use an eHealth device.

### Technology Commitment

When considering the acceptance and adaptive usage of IoT applications, objective influencing factors (e.g., actual control) and personality variables, such as the attitude toward new technologies (Scheermesser et al., 2008; Bhattacherjee and Sanford, 2009; Djamasbi et al., 2009) can be differentiated. Neyer et al. (2012) postulate technology commitment to be one of the influencing personality variables. With this construct the authors define a complex individual characteristic of dealing with technology. In particular, the openness to new technologies and the ability of a person to handle them are considered. In this context, Karrer et al. (2009) assume that technophile people are more excited about new technologies. Thus, a higher technology commitment should result in a higher usage of an innovative IoT device. However, although public acceptance of the growing technologization in healthcare is increasing (Kaltenthaler et al., 2008; Or and Karsh, 2009), some studies confirm that a negative attitude toward IoT, especially in the area of eHealth, hinders adaptation (Boonstra and Broekhuis, 2010; Li et al., 2013). Therefore, we aim to illuminate the aspect of technology commitment and examine the following research question:

Research question 2 (*RQ2*): Does technology commitment have a positive effect on the intention to use an eHealth device?

**FIGURE 1:**
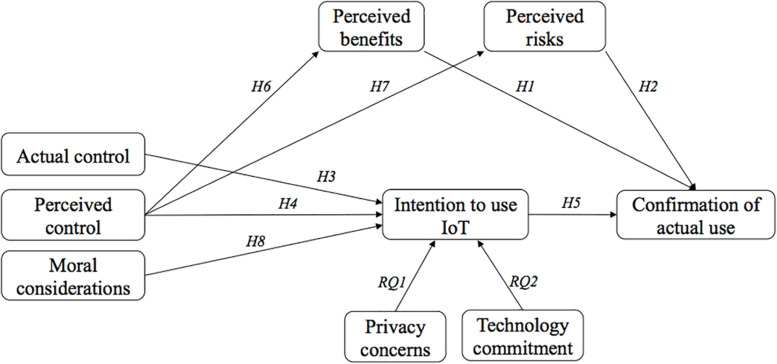
Proposed research model.

Figure 1 illustrates the proposed model of this research including all hypotheses and research questions.

## Materials and Methods

### Sample and Design

Participants were recruited through flyers and an article in the regional newspaper, advertising for a study in which they could test and evaluate a new IoT device. The device was not specified in order to raise more interest. Participants were also addressed personally and online via Facebook groups. After contacting the experimenter, they received an appointment for the laboratory study for which they received compensation in the amount of 5 euros or study credits. The ethics committee of the division of Computer Science and Applied Cognitive Sciences at the Faculty of Engineering of the University of Duisburg-Essen approved the study and written informed consent was obtained. A total of 259 people registered for appointments. Some canceled or did not have sufficient language skills to answer the questions conscientiously, resulting in an overall sample size of 209 individuals. The sample included 145 males, 63 females and eight individuals who did not specify their gender with an age range of 18–71 (*M* = 23.79, *SD* = 8.07).

**FIGURE 2:**
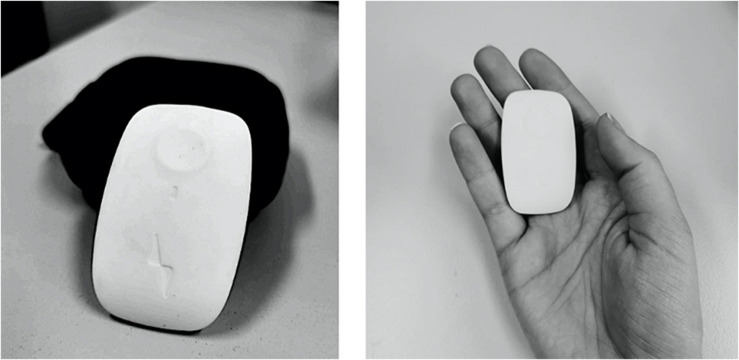
Pictures of the IoT device deployed in the study (i.e., Upright Go).

During the study, subjects tested an IoT device under real conditions. Thus, their statements regarding the intended use and the measured behavior are more valid, as they are based on experience and impressions rather than on descriptions and hypothetical assumptions. Due to the heterogeneity of IoT devices in healthcare and the various tracking techniques that differ from device to device, it is essential to focus on a specific technology in order to obtain comparable data. In addition, the device to be tested should be novel and preferably unknown technology, so that the assessment is not distorted by previous experience and subjective attitudes toward the device. Our intention was not to test a device that treats a specific disease (e.g., a blood glucose tracker for diabetes patients), but rather a device that is relevant for most people regardless of their physiological conditions and could be used in everyday life. Thus, in our study we decided to investigate a wearable healthcare device designed to improve one’s posture – the UprightGo^1^. The small gadget is placed centrally between the shoulder blades and registers the user’s posture using various sensors (see Figure 2). It detects an unhealthy posture and alerts the user by means of slight vibrations. The device can be worn permanently in order to remind the user to stand up using vibrations (training mode). Otherwise, the vibration function can be switched off and the device can be worn to quantify one’s own posture over the day (tracking mode). All data is displayed in the connected app, which can also be used to control the device. The objective of this eHealth device is to improve the posture and to prevent back pain caused by a false posture. About 95% of the participants never heard of this device before so they could only form an impression of the device during the interaction.

We employed a 3 × 1 between-subjects design manipulating the control and the amount of private data the device is able of tracking, as shown in Table 1. Participants were randomly assigned to one of the three conditions. With the exception of the possibility to control privacy settings, which is not given by this device, in our study we provided information, which corresponds to the original mode of operation of the device^2^. This approach is in accordance with the existing moral concept of an informed consent (Allhoff and Henschke, 2018). This procedure is intended to ensure that the user gives his or her consent based on information regarding the collection, processing and forwarding of personal data.

**TABLE 1:** Design of the study.

	Control	No control	
		**Privacy**	**No privacy**
Required data	Email	Age	Name
	Name	Hours sitting per day	Gender
	Gender	Information on posture and back pain Email	Body height
	Body height		Weight
	Weight		Age
	Age		Location
	Location		Hours sitting per day
	Hours sitting per day		
	Information on posture and back pain		Information on posture and back pain
Location of data storage	Online	Only on the device (local)	Online
Forwarding to third parties	Yes	No	Yes
Possibility to control privacy settings	Users can:	None	None
	• Define whether and which data the device may collect.		
	• Determine whether these data may be stored.		
	• Decide whether and for what purpose the data may be processed.		
	• Grant or withdraw permission to pass on the data to third parties.		

In order to test actual user behavior, participants were presented a cover story. In an invented newspaper article, participants were informed about a new program of German health insurance companies aiming at preventing patients’ back pain and reducing costs. Each participant in this program would receive a free wearable device from the health insurance company, which in return would require user data tracked by the device. Participants were further told that provided data would be analyzed and used to classify users into risk and non-risk patients with potential rate adjustments in healthcare insurance. Prior to the questionnaire, every participant tested the device by exploring the functions in the connected app on a laboratory smartphone and by wearing the device while sitting or standing. With the exception of few persons, most of the participants let the device on their back while filling out the questionnaire, which took about 20 min.

### Measures

After reading the newspaper article, participants were presented the descriptions of the respective device in their condition. Afterward, they were asked, whether they want to participate in the program of the health insurance company. In order to measure their behavior, participants were given the possibility to sign up for a realistic application. Confirmation of actual use of the device was therefore measured by participants’ decision to take part in the health insurance program. When participants refused to take part in the program, they could indicate their reasons.

The measurements for perceived control on a 5-point Likert-scale (from 1 = I do not agree at all to 5 = I totally agree) were adapted from Krasnova et al. (2010) and consisted of three items (e.g., I feel in control over the information I provide to the device.”). Next, components of the technology acceptance model (TAM; Davis, 1989) were assessed on a 5-point Likert-scale (from 1 = I do not agree at all to 5 = I totally agree). Basing on the TAM, we took three items for perceived ease of use (e.g., “Learning to operate the UPRIGHT GO is easy for me.”), four items for usefulness (e.g., “Using the UPRIGHT GO improves my posture.”) and three items for the intention to use the device (e.g., “I will enjoy using the UPRIGHT GO.”). Perceived privacy risk was adapted from Pavlou et al. (2007) and Hajli and Lin (2016) on a 5-point Likert-scale (from 1 = I do not agree at all to 5 = I totally agree) including items such as “I am concerned that the UPRIGHT GO is collecting too much personal information about me.” In order to assess perceived benefits, 10 items were generated based on the studies from Dienlin and Metzger (2016) and Bol et al. (2018). One example item is: “Using the UPRIGHT GO protects me from future posture problems.” Moral considerations of participants were queried using seven self-generated items (e.g., “If patients cause high costs to health insurance companies, they should also pay higher rates.”) basing on the Moral Foundations Questionnaire [MFQ20; (Graham et al., 2011)]. After confirmatory factor analysis (CFA), four items remained. Privacy concerns were assessed on a 5-point Likert-scale (from 1 = I do not agree at all to 5 = I totally agree) via 10 items (e.g., “I’m concerned that companies are collecting too much personal information about me”) developed by Smith et al. (1996), reduced to 4 items after CFA. Finally, technology commitment (Neyer et al., 2012) was assessed via 12 items on a 5-point Likert-scale (from 1 = I do not agree at all to 5 = I totally agree). One example is “I am always interested in using the latest technical equipment.” After CFA four items remained. Table 2 illustrates means, standard deviations, Cronbach’s α, McDonald’s and the average percentage of variation explained among the items for all constructs.

**TABLE 2:** Descriptive values of the constructs.

	*M*	*SD*	α	ω	AVE
Perceived control	3.42	0.98	0.87	0.87	0.7
Perceived privacy risks	2.93	1.16	0.93	0.93	0.77
Perceived benefits	4.39	0.54	0.91	0.91	0.52
Usefulness	4.21	0.66	0.87	0.88	0.64
Moral considerations	3.57	0.93	0.79	0.79	0.49
Privacy concerns	4.68	0.55	0.79	0.81	0.52
Technology commitment	4.26	0.79	0.85	0.85	0.58
Intention to use	3.84	0.97	0.89	0.89	0.73

All supplementary material including the presented scenarios, instructions and questionnaires can be viewed at https://bit.ly/3jyTHQU.

## Results

All statistical analyses were computed using the statistics software IBM SPSS Statistics 24. Bivariate correlations of the independent and dependent variables can be seen in Table 3.

**TABLE 3:** Bivariate correlations of the variables.

	1	2	3	4	5	6	7	8
(1) Actual control	–							
(2) Perceived control	−0.179**	–						
(3) Perceived risks	–0.005	−0.597***	–					
(4) Perceived benefits	0.129	0.263***	−0.167*	–				
(5) Moral considerations	–0.022	−0.158*	−0.192**	–0.032	–			
(6) Privacy concerns	0.056	–0.061	0.204**	−0.373***	0.14**	–		
(7) Technology commitment	–0.02	–0.038	0.101	0.252***	0.15**	0.219**	–	
(8) Intention to use	0.111	0.343***	−0.312***	0.591***	−0.172**	0.079	–0.030	–
(9) Confirmation of actual usage	0.54	0.242***	−0.187**	0.323***	−0.184**	0.011	–0.064	0.641***

*p < 0.05, **p < 0.01, ***p < 0.001.

In order to examine the privacy calculus within the context of IoT in healthcare, we tested whether perceived benefits of an eHealth device have a positive effect on its usage (*H1*) and whether perceived risks have a negative effect on it (*H2*). Due to the dichotomous outcome, logistic regression models were tested (Mackinnon and Dwyer, 1993; Heinze and Ploner, 2003). Both hypotheses can be accepted as the participation in the program of the health insurance in order to use the IoT device is higher when participants perceived more benefits (*B* = 1.34, *SE* = 0.35, Wald = 14.69, Odds Ratio [95% CI] = 3.82, Nagelkerke’s *R*^2^ = 0.17, *p* < 0.001) and less risks (*B* = −0.28, *SE* = 0.14, Wald = 4.00, Odds Ratio [95% CI] = 0.75, Nagelkerke’s *R*^2^ = 0.17, *p* = 0.046). In order to examine the influence of diverse benefits and to additionally validate the self-generated items used in our study, we tested the effect of usefulness (TAM; Davis, 1989) on the usage of IoT in healthcare. The results are comparable with the specific benefits used in this work (*B* = 1. 40, *SE* = 0.28, Wald = 24.60, Odds Ratio [95% CI] = 4.05, Nagelkerke’s *R*^2^ = 0.20, *p* < 0.001).

*H3* assumed that participants’ intention to use an eHealth device would be higher if they are provided with actual control of private data. To check for differences among the experimental conditions we computed a two-way analysis of variance (ANOVA). The willingness to use the IoT device does not depend on the actual control of private data collected by the device [*F*(2,206) = 1.30, *p* = 0.274, η^2^ = 0.012]. Therefore, *H3* has to be rejected.

*H4* stated that perceived control of private data has a positive effect on the intention to use an eHealth device. Results of a linear regression analysis provide support for this assumption [β = 0.34, *SE* = 0.07, *t*(207) = 5.26, *p* < 0.001] explaining 11,8% of the variance. Thus, when participants perceive control of their data, they have a higher intention of using an IoT healthcare device.

*H5* stated that participants’ intention to use an eHealth device would have a positive effect on their confirmation of actual use of the device. Due to the results of a linear regression [β = 0.31, *SE* = 0.03, *t*(207) = 12.03, *p* < 0.001], *H5* is accepted. When participants show a high intention to use IoT in healthcare, they participate significantly more in the health insurance program in order to use the eHealth device. 41,1% of confirmation of actual usage is explained by the intention to use IoT.

*H6* further assumed that perceived control of private data has a positive effect on the perception of benefits of an eHealth device. The results of a linear regression model support this assumption [β = 0.15, *SE* = 0.04, *t*(207) = 3.93, *p* < 0.001, *R*^2^ = 0.07]. Accordingly, *H6* can be accepted. *H7* referred to the negative effect of perceived control on the perception of risks. The results confirm this assumption [β = −0.71, *SE* = 0.07, *t*(207) = −10.72, *p* < 0.001] explaining 35,7% of the variance.

*H8* suggested that moral considerations have a negative effect on the intention to use an eHealth device. The data support this assumption [β = −0.18, *SE* = 0.07, *t*(207) = −2.51, *p* = 0.013, with *R*^2^ = 0.03]. The willingness to use the device is significantly lower, when people show high moral considerations.

**FIGURE 3:**
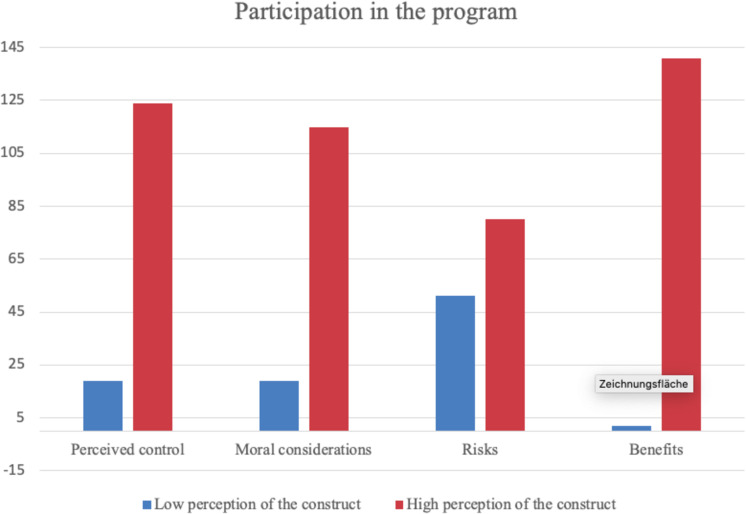
Overview of program participation in dependence of significant constructs.

Figure 3 illustrates the overall participation in the health insurance program with regard to high and low perceptions of control, risks, benefits and moral considerations. As all constructs were assessed on a 5-point likert scale, values below 2.5 are considered low while values above 2.5 are considered high.

With regard to *RQ1*, we found that participants show very high concerns regarding their privacy (see Table 2). These concerns decline when people perceive more benefits of the eHealth device. However, privacy concerns have no effect on the intention to use IoT in healthcare [β = 0.14, *SE* = 0.12, *t*(207) = 1.14, *p* = 0.256, with *R*^2^ = 0.01].

**FIGURE 4:**
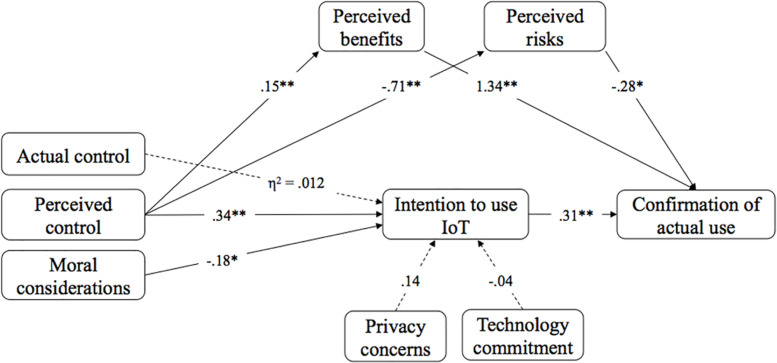
Results of the research model. Dashed lines indicate that the effect was not significant.

Finally, *RQ2* was formulated to shed light on the role of technology commitment. Although there is a positive correlation of technology commitment with perceived benefits, results of a linear regression model show no significant effects of technology commitment on the usage intention of IoT in healthcare [β = −0.04, *SE* = 0.09, *t*(207) = −0.43, *p* = 0.671, with *R*^2^ = 0.00]. The whole model is illustrated in Figure 4.

## Discussion

The current study aimed to investigate whether actual and perceived control of collected data by IoT affects the willingness to use an eHealth device given the tensions that arise in the rapidly growing eHealth sector between tracking and usage of sensitive, medical data, and individual privacy. The application of IoT in medical and health care poses the greatest risk of privacy violation compared to other sectors (Laplante and Laplante, 2016). Likewise, eHealth applications could be discriminatory for certain groups (Mittelstadt, 2017). Therefore, adaptation of IoT might be hindered especially in the area of eHealth (Boonstra and Broekhuis, 2010; Li et al., 2013). Furthermore, we examined whether privacy calculus is an appropriate theoretical approach for individuals’ decision to use IoT in healthcare and evaluated moral considerations of IoT users, such as the expectations of fair procedures from insurance companies.

### Privacy Calculus

The first two hypotheses examined the application of privacy calculus theory in the context of IoT in eHealth. More precisely, *H1* and *H2* tested whether perceived risks and anticipated benefits of an eHealth device have an effect on the confirmation of actual use of the device. Both hypotheses were approved by the results giving support to the assumption that individuals weigh risks against benefits when deciding to deploy IoT technology in the healthcare sector. Previous studies have shown that users of online services and social networking sites use the privacy calculus as the basis for the decision to disclose private information (Krasnova et al., 2012; Bol et al., 2018). The present results suggest that individuals also make this trade-off in the context of using IoT in healthcare. This is not self-evident as the way and amount of data tracking in the context of IoT applications is different from SNS environments. On the one hand, the amount of information collected by IoT exceeds the quantity of data provided online (e.g., by recording physical environmental data). On the other hand, the specific context of a health application increases the sensitivity of the data – which might further change users’ privacy-related considerations. A closer look at the results reveals that perceived benefits are more strongly related to the confirmation of actual usage of IoT in healthcare than perceived risks. This indicates that benefits of IoT applications in the healthcare sector play a primary role while privacy risks are less influential. Here, it can only be speculated that this might be due to the fact that the risks are rather abstract and not tangible for users. Accordingly, positive user expectations of the technology can override unspecific privacy risks or at least reduce their perception (Dienlin and Trepte, 2015; Hajli and Lin, 2016). In order to enable users to make more objective and reasonable decisions, it would therefore be advisable to increase the awareness of individuals regarding their privacy as well as privacy jeopardizing risks. However, the question arises whether the novelty of the device (95% of the participants were not familiar with the device) might have led to a distortion of the risk-benefit perception, due to a possible excitement of the subjects to test the new technology. In this regard, Hirschman (1980) postulates that novelty leads to a more positive attitude toward specific technology, to a higher use of this technology and to a higher intrinsic motivation to use this technology.

### Actual and Perceived Control

To begin with, the data indicate that on average participants had a rather high perception of control. We attribute this to the fact that the tested device has no video or audio sensors and the primary purpose was tracking of the back posture. Furthermore, the environment of the university laboratory may have contributed to a greater perception of control.

Related to the control of private data collected by IoT, hypotheses *H3* and *H4* assumed that both, actual control provided by device settings and the amount of control an individual perceives when using the device affect the intention to deploy IoT in healthcare. While perceived control significantly predicted usage intention, actual control did not have an effect on the willingness to use the eHealth device. This result substantiates Skinner’s (1996) distinction of actual and perceived control lending more significance on the latter as postulated by Ajzen (1991). Consequently, users of IoT in healthcare can feel insecure about the collection and handling of their data even if the device allows control by means of default settings for example. At the same time, according to the theory of planned behavior (Ajzen, 1991), they can adapt eHealth devices when they perceive control, even if actual control is not provided by the device. Especially in healthcare, this emphasizes the necessity of interventions. When users believe to be able to control their privacy even though there is no such possibility, it might be helpful to raise their awareness or to equip devices with privacy protecting features e.g., by means of governmental regulations.

The usage intention strongly correlated with participation in the health insurance program and, in line with *H5*, was able to significantly predict the registration for the program in order to use the device. In our experiment, intention to use and assessed privacy risks and benefits were based on actual interaction of participants with the device. The registration and thus the observed behavior of participants therefore have a valid foundation compared to purely hypothetical scenarios. Accordingly, based on the results of the study, we assume that individuals who test an eHealth device and intend to use it most likely also show actual usage behavior in a real situation.

Hypotheses *H6* and *H7* focused on the effect of perceived control on the perception of risks and benefits of an eHealth device. Our findings reveal that perceived control of private data leads to a higher perception of benefits and a lower assessment of privacy risks. In this context, Slovic (1987) postulates that control creates a sense of security, which might be problematic when assessing potential risks. Our results indicate that when an eHealth device does not allow any control of private data, but users still perceive control, it is likely that they trivialize the risks and underestimate potential privacy threats. At the same time, Hajli and Lin (2016) assume that users, who are less concerned about the collection of their data, have a more positive attitude toward the disclosure of personal information. This misconception might lead to a higher disclosure and a more open handling of sensitive information as scholars found that as long as users feel in control of their data and how it is handled by companies, they have a high willingness to disclose private and even identifiable information (Brandimarte et al., 2013) or share their data with third parties (Beales and Muris, 2019).

In this respect, attention should be paid to the fact that users might classify health-related data tracked by IoT in healthcare as differently sensitive. Although health data are defined as sensitive according to EU data protection legislation [Regulation (EU) 2016/679], this may imply that a person who, for example, perceives data about his or her back posture as non-sensitive might be less motivated to obtain control over this data and its utilization. Thus, this person would not be further concerned if these data were disseminated or processed. In addition to the perceived sensitivity of the data, the need for control could also be crucial in determining whether a person perceives control. According to Burger and Cooper (1979), people differ in their general desire to control the environment. This means that the perception of control may depend on whether a person wants to control a situation and therefore may be more attentive to factors that suggest control (e.g., privacy settings).

Recent studies increasingly focus on technical solutions in order to strengthen informational self-determination. In this regard, many scholars demand the consideration of privacy-by-default approaches (Kröger, 2018; Zheng et al., 2018). To be more precise, privacy-by-default means that the initial settings of IoT devices and services are adjusted to collect as little data as possible and thus to protect user’s privacy. The restriction of data collection is also addressed by article 25 of the GDPR^3^. At the same time, new privacy protection tools evolve, which actively prevent identification through IoT networks, e.g., by means of cryptography (Gu et al., 2020) or are able to detect privacy intrusion or attacks (for review on network intrusion detection systems see Chaabouni et al., 2019).

### Moral Considerations

Our last hypothesis targeted the relationship between individual’s moral considerations and the intention to use IoT in healthcare (*H8*). Our findings demonstrate that moral concerns negatively affect the intention to use eHealth technology. This means that individuals see the adaptation of IoT in healthcare as a challenge to moral values and social norms. This might be due to the difficulty of customers to assess the intentions of different stakeholders, such as insurance companies, how health related data could be used. Even if the technology itself could objectively be considered neutral, in connection with its application some may suspect an underlying strategy of data usage in the sense of data economy. Data economy means that personal data is not only gathered with the purpose for users to benefit from its analysis but that it also flows into a digital ecosystem where it can be exchanged and commercialized. As expressed by van den Hoven (2017) acceptance of technology highly depends on its ability to meet moral preferences of users. This means that, in order to assure equity and justice, access to the new technology must be made available to all and people must be able to benefit equally from it, to prevent digital divide in the health sector. However, it is precisely this sector that offers the potential to change a society based on fundamental values of solidarity. While in the pre-eHealth era, physicians and health insurances were dependent on the correct information provided by patients, IoT allows to reliably track extensive data and to draw inferences, for example on the lifestyle of an individual. As a result, the transparent patient is more vulnerable, for instance, to discriminatory profiling (Montgomery et al., 2018) or other disadvantages caused by data that IoT reveals about him or her. This might be the reason for the rather high level of moral considerations, indicated by the participants of this study. The data also revealed a negative correlation between moral consideration and perceived control. One explanation could be that people with high moral considerations have concerns regarding a fair handling of the data because they assume that other parties have control and thus the responsibility regarding the use of data. This leads to the assumption that technology is changing the health sector in such a way that users of eHealth are handing over control of the data, for example, to manufacturers or health insurance companies, while at the same time developing high expectations of a moral use of the data. This is also supported by the negative relationship between moral considerations and perceived risks. A person with high moral demands seems to assume that data use in accordance with moral standards is associated with fewer risks.

We are still at the beginning of a comprehensive technologization of the healthcare sector through IoT and standards in this area have not yet been established. This offers opportunities for a discourse between industry, health insurance companies, representatives from the health sector and ethical experts as well as governmental authorities regarding the design and implementation of eHealth with reference to moral principles. A consensus among these parties regarding the guidelines for IoT in healthcare could mean not only a broader acceptance among the population but also the protection of fundamental moral values in a society.

### Privacy Concerns

*RQ1* dealt with the investigation of a possible impact of privacy concerns on the intention to use IoT in healthcare. Our results have shown that individuals are highly concerned about their privacy. However, they are still willing to have their data tracked, if they are offered a free eHealth device, even though data collected by IoT may be interpreted in a way that is detrimental to the user. Interestingly, participants’ privacy concerns were high, at the same time, their perception of privacy risks was rather low. This could be due to the fact that the privacy concerns scale queried general privacy concerns regarding the principal handling of personal data by the device (an example item is “The device should only use personal data if authorized by the person who provided the data.”), while perceived risks rather assessed an individual’s estimation of actual risk (e.g., “I am concerned that the device collects too much personal information about me.”). Therefore, participants might be concerned about their privacy, but still do not see any concrete threat to the use of a particular eHealth device. Furthermore, perceived control, which on average was rather high, may have led to low ratings of specific risks arising from the use of the device. This again supports the assumption of Slovic (1987) regarding the negative effect of perceived control on risk estimation. Further, our findings correspond to the results of Haug et al. (2020), who postulate that even if privacy concerns are present, they do not necessarily negatively impact technology adoption. However, the authors justify these findings with trust toward and the image of manufacturers. Particularly, Haug et al. (2020) state that despite existing privacy concerns, risk perception is reduced when users of IoT have a positive impression of the manufacturer.

Another explanation for the non-significant influence of privacy concerns is that individuals have developed a fundamental skepticism toward new technologies due to the ubiquitous application of IoT, which is reflected in high worries related to one’s privacy (Fink et al., 2015; Henze et al., 2016). This skepticism seems to be present but, perhaps due to habituation, not being great enough to result in rejecting behavior toward IoT. Resignation could also be a determining factor (Wirth et al., 2018). As more and more IoT technologies enter everyday life, tracking and merging user data, it becomes impossible for an individual to follow these processes. Thus, while there is a well-founded concern for privacy, it seems to be detached from the attempt to protect it (Haug et al., 2020). This means that users accept their concerns and potential privacy risks and use IoT despite possible objections.

### Technology Commitment

Our last research question addressed the technology commitment of users and whether it leads to a higher intention to deploy IoT in healthcare. This assumption is based on considerations by Neyer et al. (2012) regarding the relationship of technology commitment as a personality variable, and the acceptance and adaptive usage of IoT applications. According to the study of Karrer et al. (2009), technophile people were expected to show a greater willingness of IoT usage. Our results did not support this assumption. Although participants’ technology commitment was high, there was no effect on the intention to use the eHealth device. However, we found a significant positive correlation of technology commitment and perceived benefits, moral considerations and privacy concerns. This indicates that individuals with a positive attitude toward IoT also perceive more benefits when deploying new technologies. However, people with a high level of technological commitment also seem to be aware of the potentially compromising capabilities of IoT, given the greater moral and privacy concerns they report. This could be mainly attributed to the health-related context.

### Limitations and Future Research

Although we conducted a lab experiment providing participants with a cover story of a health insurance program, they might have been aware of the program to be set up. However, our design allowed them to test an existing eHealth device to ensure the most realistic situation possible, which was the basis for their decision to participate in the program. Future studies should examine health related IoT usage under real circumstances e.g., by means of ambulatory assessment of patients. Another limitation of this study is the unequal gender distribution. Our sample mainly consists of females, which might have distorted the results. As prior research found gender differences in technology usage and perception of privacy, it would be interesting to shed more light on the role of gender in the context of IoT in healthcare. Finally, in our study one particular eHealth device was investigated, which challenges the generalization of our findings in relation to general use of IoT in healthcare. Additionally, it should be noted, that due to specific characteristics of this wearable device (e.g., skin contact or vibrations) participants might have felt uncomfortable using it, potentially resulting in a decreased device acceptance. Although it is important to focus on a certain kind of technology due to the heterogeneity of IoT in healthcare, future studies also need to investigate differences regarding the acceptance of diverse IoT devices from the same category, such as pedometer, sleep- or fitness tracker. To get a holistic picture, as a next step, it would be important to compare the results of investigations of different categories in order to understand why some devices are perceived differently and are more likely to be adapted than others.

## Conclusion

This research addresses the tensions that arise in the rapidly growing eHealth sector between the tracking and the usage of sensitive, medical data, and individual privacy. According to our findings, actual control of private data does not affect the willingness to use IoT in healthcare. However, individuals show a higher intention of IoT usage when they perceive to be in control of their data. Furthermore, we found that moral considerations hinder technology adaptation in this sector due to potentially discriminatory consequences of data tracking. These results show the necessity to ensure that the development and application of IoT is shaped in a morally acceptable manner. Moreover, the experiment indicates that IoT users weigh perceived risks and anticipated benefits before using an eHealth device, lending support to privacy calculus theory. Accordingly, the study extends the scope of this theoretical reasoning as the privacy calculus can also be considered in the field of eHealth expanding our understanding of eHealth technology adoption in the context of privacy.

## Data Availability Statement

The datasets generated for this study can be found in online repositories. The names of the repository/repositories and accession number(s) can be found in the article/supplementary material.

## Ethics Statement

The studies involving human participants were reviewed and approved by Ethics committee of the department of computer science and applied cognitive science, University of Duisburg-Essen. The patients/participants provided their written informed consent to participate in this study.

## Author Contributions

This manuscript describes research undertaken by EP in the framework of the Ph.D. studies, under the supervision of NCK at the University of Duisburg-Essen. Data collection, analysis, and formulation of research manuscript was undertaken by EP. NCK supervised research methodology and reviewed manuscript formulation. Both authors contributed to the article and approved the submitted version.

## Conflict of Interest

The authors declare that the research was conducted in the absence of any commercial or financial relationships that could be construed as a potential conflict of interest.

## Footnotes

1 www.uprightpose.com

2 www.uprightgo.com

3 www.eugdpr.org
